# Associations between Community Built Environments with Early Care and Education Classroom Physical Activity Practices and Barriers

**DOI:** 10.3390/ijerph18126524

**Published:** 2021-06-17

**Authors:** Bethany D. Williams, Susan B. Sisson, Dipti A. Dev, Bryce Lowery, Diane Horm, Janis Campbell, Denise Finneran, Jennifer Graef-Downard, Linda Whaley

**Affiliations:** 1 Department of Nutritional Sciences, College of Allied Health, University of Oklahoma Health Sciences Center, 1200 N. Stonewall Ave., Oklahoma City, OK 73114, USA; bethany.williams1@wsu.edu (B.D.W.); Jennifer-GraefDownard@ouhsc.edu (J.G.-D.); 2 Department of Nutrition and Exercise Physiology, Elson S. Floyd College of Medicine, Washington State University Health Sciences Spokane, 412 E Spokane Falls Blvd, Spokane, WA 99202, USA; 3 Department of Child, Youth and Family Studies, College of Education and Human Sciences, University of Nebraska-Lincoln, Louise Pound Hall, 512 N 12th St, Lincoln, NE 68588, USA; ddev2@unl.edu; 4 Department of Regional and City Planning, Gibbs College of Architecture, University of Oklahoma, 830 Van Vleet Oval, Norman, OK 73019, USA; bryce.c.lowery@ou.edu; 5 Early Childhood Education Institute, University of Oklahoma-Tulsa, 4502 E. 41st St., Tulsa, OK 74135, USA; dhorm@ou.edu; 6 Department of Biostatistics and Epidemiology, Hudson College of Public Health, University of Oklahoma Health Sciences Center, 801 N.E. 13th Street, Oklahoma City, OK 73104, USA; janis-campbell@ouhsc.edu; 7 Department of Communication Sciences and Disorders, College of Allied Health, University of Oklahoma Health Sciences Center, 1200 N. Stonewall Ave., Oklahoma City, OK 73114, USA; Denise-Finneran@ouhsc.edu; 8 Oklahoma Department of Human Services, Child Care Services, P.O. Box 25352, Oklahoma City, OK 73125, USA; Linda.Whaley@okdhs.org

**Keywords:** childcare, physical activity practices, barriers, parks, walkability, GIS

## Abstract

The influence of community-built environments on physical activity (PA) support in Early Childhood Education settings (ECEs) is unknown. The purpose of this cross-sectional study was to determine associations between community PA environments and ECE classroom PA practices. We included licensed Oklahoma ECE directors serving 3-to-5-year-old children. Parks and playground locations were exported from Google Earth. National Walkability Index was derived from 2010 US Census data. ArcMap 10.6 was used to geocode ECE locations, which were within an Activity Desert if no parks/playgrounds were located within a 1-mile radius or if Walkability Index was 10.5 or below. Classroom PA practices were determined by using the Nutrition and PA Self-Assessment tool (NAP SACC). Barriers to implementing practices were reported. Most Head Starts (*n* = 41; 80.3%), center-based childcare settings (CBC; *n* = 135; 87.0%), and family childcare homes (FCCHs; *n* = 153; 96.4%) were in an Activity Desert. Parks/playgrounds within a 10-mile buffer were correlated with classroom PA practices in FCCHs only (*p* < 0.001). Activity Desert status was not related to classroom PA practices for any ECE context (*p* > 0.029). While FCCHs may be the most vulnerable to lack of park and playground access, overall findings suggest ECEs provide a healthful micro-environment protective of the typical influence of community-built environments.

## 1. Introduction

Inadequate physical activity across the lifespan is a major public health concern [1]. Insufficient levels of physical activity in youth are specifically related to metabolic dysfunction, bone strength, fitness, and mental health [2,3,4]. Early life promotion of physical activity supports development of fine motor and social skills [5,6] and is associated with cognitive function [7] in young children. The early childhood years are formative for developing lifelong routines and habits, with physical activity patterns often continuing into adolescence and adulthood [8]. For these reasons, the US federal physical activity guidelines recommend that preschool children are physically active throughout the day, and that adult caregivers of young children encourage active play [9]. Similarly, it is recommended that young children limit daily time in sedentary behaviors, including television viewing and screen time [9]. There is much room to improve on such habits nationally, with the majority of US children consistently falling short of physical activity and sedentary time recommendations [10,11,12]. Thus, promoting healthful physical activity routines for young children has been identified as an effective strategy to improve overall national health.

Child behavior, including physical activity participation, is heavily influenced by primary caregiver encouragement and role modeling [13]. Early Childhood Education settings (ECEs) are therefore ideal for promoting behaviors that predict lifelong health of young children [14]. Such settings have promising population effect, as nearly 60% of preschoolers in the US attend out-of-home care for approximately 33−40 hours each week [15,16]. Specific practices employed in ECE settings, such as providing children with outdoor play, use of portable play equipment, teacher engagement in active play, and having sufficient indoor play space, are associated with higher levels of child physical activity [17,18]. However, some recommended practices have low implementation, and implementation varies by program demographics and ECE context (Head Starts, community-based childcare (CBC), or family childcare homes (FCCHs)) [19,20,21,22,23]. Teachers commonly report barriers to implementing classroom health practices, specifically children lacking proper outerwear, lack of indoor space for active playtime, or lack of resources to purchase play equipment [24,25,26]. There is additional need to understand prominent predictors of healthful classroom physical activity practices, especially as they may vary by ECE context.

A lack of physical activity-promoting built environment is associated with lower levels of physical activity in adults [27] and young children [28]. Specifically, physical activity is lower among those residing in areas that lack access to public parks and have poor neighborhood walkability [29]. Efforts to promote physical activity in adolescents have included promoting active transportation to and from school [30,31], which is impacted by actual and perceived physical aspects of the built environments of those school neighborhoods [32,33]. For these reasons, built environments surrounding residential areas and schools have been targeted for intervention to promote health behaviors, namely sufficient physical activity, for children of all ages and their caregivers. Influence of the built environment on children’s health and behaviors is known but less studied; this could be due to children having less autonomy to explore their neighborhood environments independently, compared to adolescents and adults. This said, little is known about the impact of the community environment on children’s caregivers and behavioral role models, specifically on ECEs policies or practices as perceived by their staff. Given that lack of indoor playspace and lack of resources to provide children with play equipment are commonly reported barriers to ECE health practice implementation [24,25,26], the surrounding community environment may play a vital supporting role for teachers promoting physical activity of their supervised children. Thus, the primary purpose of this study was to determine associations between health of community physical activity environments, including access to parks and walkability, with ECE classroom physical activity practices and barriers, specific to each ECE context (Head Starts, CBCs, and FCCHs). Ecological observation of these factors within each ECE context which experiences unique barriers to practice implementation could provide valuable information to support center- and community-specific intervention and inform tailored resources supporting teachers in adapting a health-related curriculum.

## 2. Materials and Methods

### 2.1. Study Design, Sampling Methods, and Recruitment Strategies

The Communities and Classroom Health Survey was a cross-sectional study deployed throughout the state of Oklahoma from November 2019 to February 2020 through mailed surveys distributed to licensed ECEs statewide (*N* = 2872). Locations of 343 Head Starts, 1130 CBCs, and 1648 FCCHs were obtained through a registry of licensed childcare programs provided by the Head Start Office of Collaboration and Oklahoma Department of Education. Approval was obtained from Head Start program directors before distributing surveys to centers within their program; approximately half of program directors approved of the study. ECEs affiliated with Oklahoma tribal nations with an independent Institutional Review Board were excluded from recruitment efforts. The final recruited sample included 191 Head Start centers, 1126 licensed CBCs, and 1645 licensed FCCHs. This study was not considered human subjects research by the Institutional Review Board at the University of Oklahoma Health Sciences Center.

Initial survey packets containing a cover letter, survey booklet, and a postage-paid reply envelope were mailed to all ECE settings in November 2019. A reminder postcard was mailed to all non-respondents in December 2019 and included a link for online survey participation. A second and final round of survey packets was sent to all non-respondents in January 2020. Reminder phone calls were conducted in January and February 2020. Additional survey packets were mailed as requested. An electronic link to complete surveys online by using the Research Electronic Data Capture (REDCap) secure system [34] was also distributed via email by community stakeholders in January 2020. A total of 470 surveys (23.5% response rate) were received and processed from November 2019 to February 2020; this included 64 Head Starts, 207 CBCs, 189 FCCHs, and 10 considered “Other” or ineligible (i.e., after school program only or summer camp).

### 2.2. Survey Instrument and Sample Characteristics

The Communities and Classrooms Health Survey included questions regarding ECE locations, demographics and characteristics, classroom physical activity practices, and barriers to implementation of physical activity practices (See Supplementary Materials). Surveys were completed by center directors, with instruction to answer questions for classrooms serving 3-to-5-year-old children. Directors were instructed that they could ask additional staff for help on items if they were unsure of how to respond, or if they felt another staff member (e.g., kitchen staff and teachers) might be able to provide a more accurate response. Demographics and potential covariates were reported for each ECE, including information on program context, staff responsibilities, participation in professional development, staff education, demographic distributions of children served, and information on food purchasing. These survey items were derived from a previous statewide survey in Nebraska ECEs [35].

Rural/urban status for each location was considered as a potential covariate and exploratory variable. Status was determined by using the census-tract-level 2010 secondary Rural–Urban Commuting Area (RUCA) codes [36]. RUCA codes are assigned by census tract based on that region’s population density, urbanization, and daily commuting, which identifies urban cores and economically and adjacent territories integrated with those cores. In the present study, ECE locations were geocoded by using ArcMAP 10.6 (ESRI, Redlands, CA, USA), and assigned the RUCA code representing the census tract wherein it is located. Notably, exploratory analysis revealed that constructs of the community physical activity environment surrounding ECEs did not vary significantly by urban/rural status; thus, urban/rural status was presented as a sample characteristic only.

### 2.3. Health-Enhancing Community Physical Activity Environments

Healthfulness of the community physical activity environment surrounding participating ECEs was determined in ArcMAP 10.6 (ESRI, Redlands, CA, USA) by geocoding ECE locations to determine two primary geographic constructs operationalized in four ways: (1) proximity to locations of parks and playgrounds within a 1-mile radius, (2) proximity to locations of parks and playgrounds within a 10-mile radius, (3) census tract national Walkability Index, and (4) Activity Desert construct integrating walkability and access to parks and playgrounds. Locations of parks and playgrounds were searched and exported from Google Earth for each county in Oklahoma. Each individual park and playground location was verified by using Google Streetview and up-to-date online state park listings [37,38] If the listed address was more than a 1-minute walk from the actual point location of the park or playground, latitude and longitude were recalculated using Google Earth placemark function. The original Google Earth search yielded 570 park/playground locations. Eighty-three locations were removed due to being an unsuitable play area or unrelated location; examples include playground equipment retailers, recreation management offices without park on premise, recreational vehicle (RV) sites with no park on the premises, and wildlife reserves for hunting/fishing only. An additional 58 locations were considered private park spaces requiring paid admission, and were therefore removed from analysis. Thus, 379 total parks/playground were geocoded.

The National Walkability Index was determined by using 2010 US Census Tract data, downloaded from the Environmental Protection Agency database [39,40]. Scores were calculated by the US Census based on the census block’s group-level built-environment characteristics that predict likelihood of residents walking as a mode of travel [39,41]. The characteristics of community design contributing to a higher and thus more desirable Walkability Index included (1) higher intersection density or street connectivity, (2) closer proximity to transit, (3) higher employment mix, and (4) higher housing mix. Walkability Index scores ranged from one to 20; census tracts with scores of 10.5 and below were considered “below walkable”, and tracts with scores above 10.5 were considered “walkable” [41]. To determine Walkability Index score and classification for each ECE, center locations were geocoded and assigned the Walkability Index score of their census tract.

Participating ECEs were considered as located within an Activity Desert if no parks or playgrounds were available within an accessible Euclidian distance of 1-mile, or if national Walkability Index score was “below walkable” (i.e., 10.5 or lower).

### 2.4. Classroom Physical Activity Practices and Barriers

Classroom physical activity practices were determined by using 17 survey items from the full 54-item Nutrition and Physical Activity Self-Assessment tool (i.e., NAP SACC) [19,42]. The NAP SACC is widely used, with estimates of criterion validity, inter-rater reliability and test–retest reliability previously published, indicating that the self-assessment is a stable and reasonably accurate instrument for use with childcare [19]. Items were answered on a Likert-type scale from one to four, with higher scores indicating either higher frequency or healthier degree of physical activity practice implementation. For each physical activity practice, a score of one indicated not meeting the minimum standard, two indicated meeting the minimum standard, three indicated exceeding the standard, and four indicated far exceeding the standard. Individual item scores were averaged to create five subsection scores: Active Play and Inactive Time (6 survey items), Play Environment (5 items), Supporting Physical Activity (2 items), Physical Activity Education (3 items), and Physical Activity Policy (1 item). Subgroup scores ranged from one to four, with four being the healthiest. All five subscore averages were then summed to calculate a NAP SACC Physical Activity Total Score, which ranged from five to 20.

Barriers to implementing classroom physical activity practices were derived from previous qualitative and quantitative works in the literature in ECEs of all contexts [24,25,26], and they were approved by an interdisciplinary scientific advisory team. Specifically, there were 16 items to determine barriers to promoting physical activity for young children. Examples of practices to promote physical activity were specified on the survey, and included providing indoor and outdoor playtime, talking with children about physical activity, providing teacher-led physical activity, providing indoor and outdoor play space and equipment, and verbally and physically encouraging children to be physically active. Providers were asked to report “yes” or “no” to whether their ECE experienced each potential barrier.

### 2.5. Statistical Analysis

Descriptive statistics (means, standard deviations, and frequencies) were calculated, and all analyses were performed, in SAS v. 9.4. (SAS Institute, Inc., 2013, Carey, NC, USA). All analyses were performed separately for each ECE context (Head Starts, CBCs, and FCCHs). The Shapiro–Wilk test for normality indicated that primary outcome data were not normally distributed (*p* < 0.05 for all ECE contexts). Kruskal–Wallis one-way analysis of variance was used to determine differences in ECE classroom physical activity practice scores between Head Starts, CBCs, and FCCHs. Fisher’s Exact test was used to determine differences in prevalence of reporting barriers (“%yes”) to implementing ECE classroom physical activity practices between Head Starts, CBCs, and FCCHs.

To address the primary aims of the present study, Spearman rank order correlation was used to determine correlation between continuous characteristics of the community physical activity environment (i.e., number of parks within radius buffer and Walkability Index) with ECE classroom physical activity practices. Wilcoxon Rank Sum test was used to determine differences in ECE classroom physical activity practice scores between those located within an Activity Desert and those located within a Non-Desert. Fisher’s Exact test was used to determine differences in prevalence of reporting barriers (“%yes”) to implementing ECE classroom physical activity practices between those located within an Activity Desert and those located within a Non-Desert. The Benjamini Hochberg correction was applied to primary analyses to account for multiple comparison and control for False Discovery Rate, with adjusted alpha *p* < 0.029.

## 3. Results

In total, 474 Oklahoma ECEs responded, with final response rates being 33.5% for Head Starts (*n* = 64), 18.2% for CBCs (*n* = 206), and 11.6% for FCCHs (*n* = 192). ECEs were excluded if they indicated they were a “Public Pre-K”, did not report ECE context (*n* = 15; 3.1%), or had missing data on primary variables of interest (*n* = 94; 19.8%). Thus, the final analytic sample comprised 365 ECEs, including 51 Head Starts, 155 CBCs, and 159 FCCHs (Table 1).

For all ECE contexts, the majority participated in CACFP (76.9%), whereas fewer participated in programs to enhance physical activity behaviors, i.e., Go NAP SACC (3.5%) and Healthy Body, Healthy Minds (4.1%). Compared with FCCHs, Head Start centers and CBCs serve more children total and more 3-to-5-year-old children, and reported having a higher number of teachers and additional supporting staff (Table 1). Compared with CBCs and FCCHs, Head Start centers reported highest prevalence of teachers with a Bachelor’s degree or higher, and served a more diverse racial/ethnic distribution of young children. Further, Head Start centers reported the lowest prevalence of regularly engaging in out-of-center activities, had the fewest parks located in a 10-mile buffer radius, had the highest prevalence of being located in a “walkable” census block group, and had the lowest prevalence of being classified within an Activity Desert. Compared with Head Starts and FCCHs, CBCs demonstrated the highest number of parks located in a 10-mile buffer radius and the highest average Walkability Index. Finally, compared with Head Starts and CBCs, FCCHs reported the highest prevalence of regularly engaging in out-of-center activities, the lowest number of parks located within each specified buffer radius, the lowest average Walkability Index, and the highest prevalence of being located within an Activity Desert.

### 3.1. ECE Context and Classroom Physical Activity Practices

Classroom physical activity practices and reported barriers to implementing those practices significantly varied by ECE context (Table 2).

Across all ECE contexts, average subscores for classroom physical activity practices were mostly higher than two, indicating that Oklahoma ECEs were meeting minimum recommended standards, for the most part. Head Start centers demonstrated the highest NAP SACC Physical Activity Total Score, with the highest scores for Supporting Physical Activity, Physical Activity Education, and Physical Activity Policy. FCCHs demonstrated the lowest NAP SACC Physical Activity Total Score and the lowest values for many subscores. However, Head Start centers reported healthier practices for Physical Activity Education than did CBCs. Subscores for Active Play and Inactive Time and Play Environment were the highest subscores overall across all ECE contexts.

The most commonly reported barriers to implementing classroom physical activity practices across contexts included limited room for indoor playtime, undesirable weather conditions for outdoor play, and children arriving wearing improper clothing. Head Starts reported lower prevalence of all barriers than did CBCs and FCCHs. Compared with Head Starts and FCCHs, CBCs reported the highest prevalence of barriers, including providers being unsure how to encourage child physical activity, limited space for storing play equipment, and licensing limits for type of play equipment allowed. Compared with Head Starts and CBCs, FCCHs reported the highest prevalence of lack of resources to purchase play equipment.

### 3.2. Parks/Playgrounds, Walkability, Activity Deserts, and Classroom Physical Activity Practices

Constructs of the community physical activity environment, including number of parks or playgrounds within a 1- and 10-mile buffer radius and average Walkability Index, were not correlated with classroom physical activity practices in Head Starts or CBCs (*p* > 0.029 for all) (Table 3).

Number of parks located within a 10-mile buffer radius was correlated with NAP SACC Physical Activity Total Score, and specifically the Supporting Physical Activity subscore, in FCCHs only (*p* < 0.001 for both). Classroom physical activity practice scores and barriers did not differ by Activity Desert status across all ECE contexts (Table 4; *p* > 0.029 for all).

## 4. Discussion

The present study aimed to determine how the community physical activity environment, including presence of surrounding parks/playgrounds, Walkability Index, and Activity Desert status, was associated with ECE classroom physical activity practices and barriers by ECE context (Head Starts, CBCs, and FCCHs). Across all ECE contexts, the majority (72–82%) regularly engaged in out-of-center community activities, few were located within a “walkable” area (9–24%), and even fewer were considered to be in a Non-Desert (4–20%). However, constructs of surrounding community physical environments varied across ECE contexts; specifically, FCCHs had fewer nearby parks and playgrounds, were least likely to be located within a “walkable” census block group, and were most likely to be located within an Activity Desert. Present findings were consistent with similar studies in observing that Head Start centers report the healthiest frequency/degree of classroom physical activity practices, while FCCHs typically demonstrate the lowest [19,20,21,22,23]. Finally, the present study found that the presence of more parks and playgrounds was related to healthier physical activity classroom practices in FCCHs only. Activity Desert status was not related to classroom physical activity practices and barriers among any ECE context. To our knowledge, this is the first study to report on how physical activity built environments surrounding ECEs relate to classroom physical activity practices and related experiences (i.e., perceived barriers) of ECE staff.

Overall, the majority of participating ECEs lacked access to parks and playgrounds within a radius of 1 mile. Average Walkability Index of ECE locations were scored below what is considered “walkable”. To our knowledge, this is the first study to describe constructs of the community physical activity environment surrounding ECEs in a statewide sample. Access to public parks and play areas, as well as actual and perceived walkability and neighborhood safety, are related to higher physical activity levels, lower prevalence of obesity, and higher likelihood of active transportation use for residential communities [27,28,30,31]. Specifically, physical activity is lower among those residing in areas that lack access to public parks and have poor geographic walkability [29]. Notably, in the 2017 US Report Card on Walking and Walkable Communities, Oklahoma ranked poorly as one of just 14 states that met none of the six defined standards supporting “walkable” communities [43]. This lack of access to healthful community physical activity environments may contribute to low reporting of regular community engagement observed in the present study sample across all ECE contexts. Future studies could therefore benefit from describing how ECE community environments may differ across various regions of the US, and additionally understanding how those environments shape ECE community engagement and related outdoor health practices. Such findings could provide important insight into how ECEs engage with their surrounding environments and whether this engagement and influence differs by state/regional context.

The present findings were consistent with similar studies in observing that Head Start centers report healthiest frequency/degree of classroom physical activity practices and FCCHs report the lowest [19,20,21,22,23,35,44]. Practices with the largest difference in implementation between Head Starts and other ECE contexts were physical activity education provided for staff and parents, and the presence of a physical activity policy. Studies have previously reported that classroom health practices are more desirable when staff complete regular continued education [45,46] and when center- or state-level policy includes those practices [47,48]. However, physical activity training is not typically pursued by CBC and FCCH staff [49,50,51], and related classroom practices are not typically emphasized in state licensure policy [52,53]. In comparison, Head Starts adhere to performance standards that are typically much higher than those expected by state licensing; requirements include frequent staff education and the presence of a stringent policy promoting children’s health behavior. The present study in combination with previous findings suggests a need to promote educational opportunities and policy focused on child physical activity promotion for ECE classrooms. Such changes could be effective strategies to promote overall classroom health practices, especially for CBCs and FCCHs. These consistent differences in classroom health practices between ECE contexts additionally highlight the importance of understanding their predictors, specific to each facility type.

For FCCH providers only, number of nearby parks and playgrounds were associated with classroom physical activity practices. Across all ECE contexts, classroom physical activity practices did not differ by Activity Desert status. Thus, the overall results indicate that ECE programs, especially Head Starts and CBCs, are protective of the typical influence of built environments on health practices for young children. However, FCCHs may be more vulnerable to influence of their surrounding community physical activity environments, particularly in regards to practices supporting physical activity for young children. Contrary to the author’s hypothesis, number of parks within 10 miles, but not within 1 mile, was related to FCCH classroom physical activity practice scores. This may be due to childcare providers utilizing community resources, such as parks, that are not necessarily within walking distance of their center’s location. It is also possible that within larger neighborhoods, a high density of parks are correlated with neighborhood socioeconomical demographics, perceived safety, community resources and engagement, or other potential confounders. Regardless, this association being significant in FCCHs only may be attributable to higher reported frequency of community engagement, combined with FCCHs most commonly reporting lack of resources to purchase toys and play equipment and therefore depending on public play spaces to encourage child physical activity. Recent findings also show Head Starts and CBCs devoting resources to outsourcing companies (i.e., adult organized youth sports leagues) to encourage physical activities for children in their care, a practice likely not feasible among FCCHs [54]. Across the literature, FCCHs report the lowest implementation of physical activity practices [49,50,51,55,56], and are less likely to have a written physical activity policy [49] than are other ECE contexts. There is additional evidence suggesting that children attending FCCHs are at higher risk of obesity than are those attending Head Starts and CBCs [57]. Given these concerns, the present study suggests a potential mechanism to improve FCCH classroom practices, and the health of those children served, through constructing tailored resources for FCCH providers in low-access communities. Promoting physical activity policy in FCCHs, and specifically encouraging teacher-led physical activity strategies to overcome limitations in space or play equipment, may be necessary next steps to improve health of these settings.

Strengths and limitations of the present study should be considered. Strengths included use of geographic data, i.e., locations of parks and playground, which were obtained at the same time as the survey was employed and validated by an online search for each individual site. National Walkability Index is a validated measure of the community built environment, and calculated by the US Census [39,40]. The present study also used a statewide sample representing each of the three primary ECE contexts. The primary study aims are novel, and provide valuable insight to inform future policy development and evaluation for ECE settings located in low-access neighborhoods. The present study was also subject to limitations. First, due to the cross-sectional study design, causality cannot be inferred. The current available National Walkability Index was scored from 2010 Census data, and therefore may not fully represent community physical activity environments surrounding ECEs at the time the statewide survey was distributed in 2019/2020. Data were self-reported by ECE directors and could be subject to social desirability, particularly among Head Start centers with assumed knowledge of standards and best practices. The current study sample may also be subject to selection bias, and sample sizes were somewhat limited in stratified analyses. Similarly, findings in the present study may be specific to the state of Oklahoma, having unique geography, demographics and culture. Therefore, the findings should be interpreted with caution and may have limited external generalizability. Finally, the primary respondent was the ECE program director, who may not have complete knowledge of current classroom activities or provider barriers. However, respondents were instructed to defer to the staff with most accurate insight on that practice. Further, the NAP SACC tool to assess classroom health practices is widely used and has been validated against practices observed multiple days in-classroom by trained research personnel [19].

## 5. Conclusions

The present study provides important insight into how the surrounding community is related to ECE center classroom practices, which is essential to inform tailored resources and policy change to promote physical activity and health for young children. Participating Oklahoma ECEs were mostly located within environments with a lack of access to parks and playgrounds and with poor walkability. Overall, classroom practices and barriers were not associated with constructs of the community physical environment, and were not different by Activity Desert status. However, in FCCHs, higher numbers of nearby parks and playgrounds within a 10-mile proximity were related to healthier overall classroom physical activity practices. These findings suggest that Head Starts and CBCs provide a healthful micro-environment that is protective of the typical influence of the built environment, which may be particularly important for those children served who lack access to health resources in their residential communities. However, FCCHs may be more vulnerable to the health of their surrounding communities, in part due to a lack of resources for providers to purchase sufficient play equipment to promote child health. Future studies should consider the following: (1) describing how ECE community physical activity environments may differ across various regions of the US; (2) understanding how those community physical activity environments shape ECE community engagement, outsourcing companies for physical activity promotion, and related outdoor health practices; (3) constructing tailored resources to promote classroom health for FCCH providers in low-access communities; and (4) determining strategies to promote physical activity policy in FCCHs. Such findings could provide important insight for scientists, practitioners, and policy-makers on how ECEs engage with their surrounding environments, and how to improve health practices for those most vulnerable to those low-access environments.

## Figures and Tables

**Table 1:** Oklahoma Early Care and Education programs participating in the Communities and Classroom Health Survey in 2019/2020, by context (*n* = 365).

	Head Start (*n* = 51)	CBC (*n* = 155)	FCCH (*n* = 159)
Center Hours (n (%))			
Half Day	16 (31.3)	15 (9.6)	7 (4.4)
Full Day	41 (80.3)	145 (93.5)	154 (96.8)
“Other”	0 (0.0)	10 (6.4)	3 (1.8)
Number of Teachers (mean ± SD)	4.3 ± 5.2	4.3 ± 4.4	1.4 ± 0.8
Percent of Teachers with Bachelor’s Degree or Higher (mean% ± SD)	18.7 ± 31.3	9.7 ± 21.9	11.3 ± 29.6
Number of Additional Supporting Staff (mean ± SD)	4.9 ± 7.3	2.1 ± 2.2	0.65 ±0.8
Number of Total Classrooms (mean ± SD)	4.7 ± 5.0	6.1 ± 3.3	1.5 ± 1.5
Number of Classrooms for 3–5-Year-Olds (mean ± SD)	3.5 ± 4.8	2.2 ± 1.1	1.3 ± 1.4
Number of Total Children (mean ± SD)	64.7 ± 82.6	66.3 ± 45.0	9.0 ± 4.1
Number of 3-to-5-Year-Old Children (mean ± SD)	56.1 ± 83.7	26.5 ± 19.9	3.8 ± 2.5
Percent of 3-to-5-Year-Old Children who are the following ethnicities: (mean% ± SD)			
Hispanic	16.4 ± 18.0	4.8 ± 6.5	3.9 ± 11.4
American Indian	17.4 ±21.8	12.8 ± 17.4	14.2 ± 25.3
Asian	1.9 ± 7.5	1.4 ± 4.2	0.7 ± 4.9
Black or African American	11.3 ± 14.1	10.5 ± 18.3	10.3 ± 23.4
Native Hawaiian or Pacific Islander	1.1 ± 6.7	0.5 ± 1.9	0.8 ± 7.3
White or Caucasian	43.4 ± 26.3	55.4 ± 30.8	57.2 ± 38.2
Mixed race	10.9 ± 11.6	8.4 ± 13.0	9.7 ± 20.5
Other	0.7 ± 5.6	0.3 ±2.3	0.1 ± 1.3
Non-specified	0.5 ± 3.7	8.1 ±23.2	5.3 ± 18.4
NAEYC Accredited (*n* (%))	12 (24.0)	17 (11.1)	11 (6.9)
Professional Program Participation (*n* (%))			
CACFP	50 (98.0)	91 (58.7)	140 (88.0)
Go NAP SACC	4 (7.8)	5 (3.2)	4 (2.5)
Healthy Body, Healthy Minds	3 (5.8)	7 (4.5)	5 (3.1)
Happy Healthy Homes	2 (3.9)	0 (0.0)	10 (6.2)
Certified Early Childhood	11 (21.5)	20 (12.9)	11 (6.9)
Out-of-Center Community Engagement (*n* (%))			
Very often or Somewhat often	9 (17.6)	42 (27.3)	40 (25.2)
Not very often or Never	42 (82.2)	111 (72.4)	118 (74.6)
Health Advisory Committee (*n* (%))			
Yes	37 (72.5)	22 (14.1)	8 (5.0)
No	8 (15.6)	129 (83.2)	145 (91.7)
Not sure	6 (11.7)	4 (2.5)	5 (3.1)
Presence of Outdoor Play Policy (*n* (%))			
Yes, Oklahoma Childcare Licensing	44 (86.2)	115 (74.1)	118 (74.2)
Yes, Plus Additional Policy	3 (5.8)	31 (20.0)	17 (10.6)
No	4 (7.8)	9 (5.8)	24 (15.0)
Percent Urban/Rural within Census Tract (*n* (%))			
Urban	24 (47.0)	96 (61.9)	96 (60.3)
Rural	27 (52.9)	59 (38.0)	63 (39.6)
Number of Parks/Playgrounds (mean ± SD)			
Within 1 mile	0.7 ± 1.1	0.5 ± 0.9	0.2 ± 0.7
Within 5 miles	3.4 ± 3.7	3.7 ± 3.1	2.7 ± 2.6
Within 10 miles	5.3 ± 5.0	7.4 ± 5.0	6.8 ± 5.5
Presence of Parks/Playgrounds in Buffer (*n* (%))			
≥1 Within 1 mile	20 (39.2)	49 (31.6)	28 (17.6)
≥1 Within 5 miles	36 (70.5)	130 (83.8)	127 (79.8)
≥1 Within 10 miles	42 (82.3)	141 (90.9)	140 (88.0)
Neighborhood Walkability Index (mean ± SD)	7.9 ± 2.9	8.5 ± 2.4	7.2 ± 2.3
Classification of Neighborhood Walkability (*n* (%))			
Below Average Walkability (≤10.5)	39 (76.4)	120 (77.4)	145 (91.1)
“Walkable” (>10.5)	12 (23.5)	35 (22.5)	14 (8.8)
PA Desert Status by Urban/Rural (*n* (%))			
Urban, PA Desert	22 (43.1)	90 (58.0)	92 (57.8)
Urban, Non-Desert	2 (3.9)	6 (3.8)	4 (2.5)
Rural, PA Desert	19 (37.2)	45 (29.0)	61 (38.6)
Rural, Non-Desert	8 (15.6)	14 (9.0)	2 (1.2)

CBC = community-based childcare; FCCH = family childcare home; NAEYC = National Association for the Education of Young Children; CACFP = Child and Adult Care Food Program (CACFP) by USDA; Go NAP SACC = Nutrition and Physical Activity Self-Assessment for Child Care.

**Table 2:** Classroom physical activity practice scores and barriers among Oklahoma ECE programs participating in the Communities and Classroom Health Survey in 2019/2020, by childcare context.

	Head Start (*n* = 51)	CBC (*n* = 155)	FCCH (*n* = 159)	*p*-Value
Classroom Physical Activity Practice Scores (mean ± SD)				
NAP SACC Physical Activity Total Score	17.1 ± 2.1	14.9 ± 2.5	13.6 ±2.7	<0.0001 *
1. Active Play and Inactive Time Score	3.1 ± 0.3	3.2 ± 0.4	3.0 ± 0.4	0.0252 *
2. Play Environment Score	3.6 ± 0.3	3.5 ± 0.4	3.3 ± 0.4	<0.0001 *
3. Supporting Physical Activity	3.6 ± 0.0	3.0 ± 0.7	2.8 ± 0.7	<0.0001 *
4. Physical Activity Education	3.2 ± 0.8	2.1 ± 0.9	2.5 ± 0.8	<0.0001 *
5. Physical Activity Policy	3.5 ± 0.9	2.6 ± 1.2	2.1 ± 1.2	<0.0001 *
Barriers to Classroom Physical Activity Practices (%yes)				
Competing curriculum priorities over PA.	9 (17.6)	31 (17.4)	19 (10.9)	0.1888
Providers unsure how to encourage child PA.	1 (1.9)	20 (12.9)	11 (6.9)	0.0337 *
Limited space for storing toys/equipment.	11 (21.5)	63 (40.9)	58 (36.4)	0.0411 *
Lack of resources to purchase toys/equipment.	10 (19.6)	63 (40.9)	69 (43.4)	0.0067 *
Limited room for indoor active playtime.	26 (50.9)	73 (41.0)	76 (43.9)	0.4478
Limited room for outdoor playtime.	3 (5.8)	14 (7.9)	17 (9.9)	0.6772
Undesirable weather conditions limiting PA.	29 (56.8)	85 (55.5)	102 (28.1)	0.1826
School board does not support PA promotion.	3 (5.8)	6 (3.4)	3 (1.7)	0.2462
Parents/guardians do not support PA promotion.	4 (7.8)	8 (4.5)	6 (3.4)	0.4350
Children arrive wearing improper clothing for PA.	21 (41.1)	69 (38.7)	66 (38.1)	0.9185
Provider concern for child injury.	3 (5.8)	18 (10.1)	16 (9.2)	0.7206
Provider concern for neighborhood safety.	3 (5.8)	5 (2.8)	10 (5.8)	0.3060
Licensing limits type of play equipment allowed.	2 (4.0)	29 (16.5)	19 (11.0)	0.0441 *
Providers prefer to partake in sedentary activity.	3 (5.8)	21 (11.8)	10 (5.7)	0.1155
Providers feel playtime with children is stressful.	1 (1.9)	13 (7.3)	9 (5.2)	0.3757

* indicates significant difference among groups (*p*-value < 0.05). CBC = community-based childcare; FCCH = family childcare home; NAP SACC = Nutrition and Physical Activity Self-Assessment for Child Care; PA = physical activity; ECE = Early Care and Education.

**Table 3:** Associations between classroom physical activity practice scores with constructs of the community physical activity environment among Oklahoma ECE programs participating in the Communities and Classroom Health Survey in 2019/2020, by childcare context.

	Head Start (*n* = 51)			CBC (*n* = 155)			FCCH (*n* = 159)		
	# Parks within 1 mile	# Parks within 10 miles	Nat’l. Walk. Index	# Parks within 1 mile	# Parks within 10 miles	Nat’l. Walk. Index	# Parks within 1 mile	# Parks within 10 miles	Nat’l. Walk. Index
NAP SACC Physical Activity Total Score	0.05	−0.10	0.27	−0.08	−0.05	0.05	0.08	0.28 *	0.10
1. Active Play and Inactive Time Score	0.05	−0.25	0.04	−0.05	−0.10	−0.08	0.01	0.15	0.02
2. Play Environment Score	−0.07	0.00	0.25	−0.07	0.03	0.02	0.01	0.16	0.11
3. Supporting Physical Activity	0.01	−0.01	0.33	−0.14	−0.01	0.00	−0.03	0.27 *	0.12
4. Physical Activity Education	−0.07	0.01	0.18	−0.09	−0.05	0.04	0.00	0.21	−0.01
5. Physical Activity Policy	0.10	−0.01	0.16	0.01	−0.01	0.12	0.13	0.14	0.09

Spearman rank order correlation statistics are presented. * A *p*-value < 0.029; indicates significant association after Benjamini Hochberg correction for False Discovery Rate. CBC = community-based childcare; FCCH = family childcare home; NAP SACC = Nutrition and Physical Activity Self-Assessment for Child Care; Nat’l. Walk. Index = National Walkability Index.

**Table 4:** Differences in classroom physical activity practice scores and barriers based on Activity Desert status among Oklahoma ECE programs participating in the Communities and Classroom Health Survey in 2019/2020, by childcare context.

	Head Start (*n* = 51)		CBC (*n* = 155)		FCCH (*n* = 159)	
	Activity Desert (*n* = 41)	Non-Desert (*n* = 10)	Activity Desert (*n* = 135)	Non-Desert (*n* = 20)	Activity Desert (*n* = 153)	Non-Desert (*n* = 6)
Classroom Physical Activity Practice Scores (mean ± SD)						
NAP SACC Physical Activity Total Score	17.1 ± 2.1	17.7 ± 1.9	14.9 ± 2.5	14.8 ± 2.8	13.4 ± 2.7	14.0 ± 2.1
1. Active Play and Inactive Time Score	3.1 ± 0.3	3.2 ± 0.4	3.2 ± 0.4	3.0 ± 0.3	3.0 ± 0.4	3.1 ± 0.3
2. Play Environment Score	3.6 ± 0.3	3.6 ± 0.2	3.5 ± 0.4	3.4 ± 0.3	3.3 ± 0.4	3.4 ± 0.1
3. Supporting Physical Activity	3.5 ± 0.6	3.7 ± 0.5	3.0 ± 0.6	2.9 ± 0.8	2.8 ± 0.7	2.6 ± 0.4
4. Physical Activity Education	3.3 ± 0.6	3.3 ± 0.9	2.5 ± 0.8	2.3 ± 0.9	2.1 ± 0.9	1.9 ± 0.7
5. Physical Activity Policy	3.4 ± 0.9	3.8 ± 0.6	2.6 ± 1.2	3.0 ± 1.1	2.0 ± 1.2	2.8 ± 1.3
Barriers to Classroom Physical Activity Practices (%yes)						
Competing curriculum priorities over PA.	8 (19.5)	2 (20.0)	20 (14.8)	4 (20.0)	15 (9.8)	1 (16.6)
Providers unsure how to encourage child PA.	1 (2.4)	0 (0.0)	17 (12.5)	3 (15.0)	10 (6.5)	1 (16.6)
Limited space for storing toys/equipment.	9 (21.9)	2 (20.0)	58 (42.9)	5 (26.3)	58 (37.9)	0 (0.0)
Lack of resources to purchase toys/equipment.	8 (19.5)	2 (20.0)	52 (38.5)	11 (55.0)	67 (43.7)	2 (33.3)
Limited room for indoor active playtime.	22 (53.6)	5 (50.0)	57 (42.2)	7 (35.0)	70 (45.7)	3 (50.0)
Limited room for outdoor playtime.	1 (2.4)	2 (20.0)	10 (7.4)	3 (15.0)	16 (10.6)	0 (0.0)
Undesirable weather conditions limiting PA.	24 (58.4)	5 (50.0)	74 (55.2)	11 (57.8)	98 (64.0)	4 (66.6)
School board does not support PA promotion.	2 (4.8)	1 (10.0)	5 (3.8)	1 (5.2)	2 (1.3)	0 (0.0)
Parents/guardians do not support PA promotion.	4 (9.7)	0 (0.0)	5 (3.8)	2 (10.0)	4 (2.6)	0 (0.0)
Children arrive wearing improper clothing for PA.	16 (39.0)	5 (50.0)	54 (40.0)	8 (40.0)	58 (37.9)	1 (16.6)
Provider concern for child injury.	2 (4.8)	1 (10.0)	12 (8.9)	4 (20.0)	13 (8.5)	0 (0.0)
Provider concern for neighborhood safety.	2 (4.8)	1 (10.0)	3 (2.2)	1 (5.0)	10 (6.5)	0 (0.0)
Licensing limits type of play equipment allowed.	2 (4.8)	0 (0.0)	18 (13.6)	7 (35.0)	15 (9.8)	1 (16.6)
Providers prefer to partake in sedentary activity.	3 (7.3)	1 (10.0)	16 (11.8)	3 (15.0)	10 (6.5)	0 (0.0)
Providers feel playtime with children is stressful.	1 (2.4)	0 (0.0)	9 (6.6)	3 (15.0)	8 (5.2)	0 (0.0)

A *p*-value < 0.029; indicates significant association after Benjamini Hochberg correction for False Discovery Rate. ECE = center for Early Childhood Education; CBC = community-based childcare; FCCH = family childcare home; NAP SACC = Nutrition and Physical Activity Self-Assessment for Child Care; PA = physical activity.

## Data Availability

The data presented in this study are available upon request from the corresponding author. The data are not publicly available, due to the need to maintain the confidentiality of participating ECE programs.

## Supplementary Materials

The following are available online at https://www.mdpi.com/article/10.3390/ijerph18126524/s1, Communities and Classroom Health Survey.
