# Supplementary material for: Associations between Community Built Environments with Early Care and Education Classroom Physical Activity Practices and Barriers
Source: Int J Environ Res Public Health. 2021 Jun 17;18(12):6524. doi: 10.3390/ijerph18126524 (PMC8296338; doi:10.3390/ijerph18126524)
Supplement: Supplementary file 1 [file ijerph-18-06524-s001.zip › ijerph-1241569-supplementary.pdf]

Dear ECE center/home director,

We are conducting a research study seeking to better understand how Early Childhood Education (ECE) centers' surrounding community environment may influence classroom health practices and/or experiences of the childcare staff. Results from this study will provide valuable information to support center-specific intervention and implementation of tailored resources to provide support for teachers who have difficulty implementing health-based curriculum. We are requesting your participation as a center director of a licensed ECE center that **includes children ages 3 to 5 years old**. For this study, **a "center" may be a Head Start center, community-based childcare center, or family childcare home.**

This survey contains questions about your ECE center's location and demographics; nutrition, physical activity and other health-related practices; and barriers experienced when attempting to implement classroom health practices. We estimate that it will take about 25 minutes to complete the survey. If you complete the survey, you will be given the opportunity to enter into a drawing for one of forty-five $25 Amazon gift cards.

Your participation in this study is voluntary. If you choose not to participate or choose to participate but then withdraw from this study, there will be no penalty to you. You can stop the survey at any time. The attached survey will be confidential.

If you have any questions concerning this research study, please email the Principal Investigator, Dr. Susan Sisson, at susan-sisson@ouhsc.edu (405-271-2113 x 41176) or contact the Study Coordinator, Bethany Williams, at Bethany-Williams@ouhsc.edu (405-271-2113 x 41173).

COMMUNITIES AND CLASSROOM HEALTH SURVEY

INSTRUCTIONS: We ask the survey be filled out by **one** director or provider most familiar with the ECE's nutrition, physical activity and other health-related practices. However, you may come across questions that you think someone else in your ECE could answer more easily than you. If so, **please feel free to ask your staff for help**.

- These questions ask about health practices in your **preschool-age classroom(s) (3-5 year old) or Head Start program**. Please answer questions only about your preschool classroom with children ages 3-5 years. Unless otherwise specified, please do NOT include information about your infant classroom or your Early Head Start program (if you have one).
- When we refer to teachers, we mean individuals who have direct contact with preschoolers (3-5 years) and are responsible for supervising meals or snacks for preschool children.
- Please answer about what is currently happening in your ECE, unless a question asks about another time period.
- We do not expect you or your staff will have to consult any administrative records in order to complete the survey.
- If you are unsure about how to answer a question, please give the best answer you can rather than leaving it blank.

For your convenience, you may either complete and mail back the surveys contained in this packet using the postage-paid envelope provided, OR you may instead complete the surveys online. **PLEASE USE THE FOLLOWING LINK IF YOU WOULD LIKE TO COMPLETE THE SURVEYS ONLINE: https://is.gd/OKcommunities_classrooms**

First, to determine ECE access to healthful community resources (healthful food outlets, parks, playgrounds, etc.) we will need the **name and physical location (address)** of your ECE center. This information will remain confidential, and findings will only be discussed in combination with data from other ECEs.

____

____

ABOUT YOUR CENTER

1. Which of the following best describes your center?

	YES	NO
Center based child care	◯	◯
Family childcare home	◯	◯
Head Start	◯	◯
Public pre-k program	◯	◯
Other (*please specify*): ______________________	◯	◯

2. Which of the following best describes your center (*select all that apply*)?

- ☐ Half-day
- ☐ Full-day
- ☐ Other (*please specify*):

____

3. Is your center NAEYC accredited?

◯ Yes
◯ No
◯ Not sure

4. Is your center tribally-affiliated?

◯ Yes
◯ No
◯ Not sure

5. Is your center fully enrolled at this time?

◯ Yes
◯ No
◯ Not sure

If you answered "No", what do you think is the major reason?

____

6. Do 3-5 year old children often leave your center to enroll in other programs (public pre-K, other preschools, etc.)?

◯ Yes
◯ No
◯ Not sure

If you answered "Yes", what do you think is the major reason?

____

7. What is the number of teachers employed at your center who serve 3-5 year old children?

____

8. Of the teachers employed at your center who serve 3-5 year old children, what estimated percentage have a Bachelor's degree or higher?

____

9. What is the number of additional supporting staff employed at your center (assistant teachers, aids, substitutes, etc.) who serve 3-5 year old children?

____

10. What is the number of classrooms in your center **total**?

____

11. What is the number of classrooms in your center **with children who are 3-5 years old**?

____

12. What is the number of children in your center **total**?

____

13. What is the number of children in your center **who are 3-5 years old**?

____

14. On a typical day, what estimated percentage of 3-5 year old children in your center are Hispanic or Latino?

____

15. On a typical day, what **estimated percentage** of 3-5 year old children in your center are of the following racial backgrounds?

______ American Indian or Alaska Native

______ Asian

______ Black or African American

______ Native Hawaiian or Pacific Islander

______ White or Caucasian

______ Mixed race

______ Other (*please specify*):

____

16. To your knowledge, what **estimated percentage** of 3-5 year old children in your center:

…participate in the Supplemental Nutrition Assistance Program (SNAP)?

____

…participate in the Special Supplemental Nutrition Program for Women, Infants, and Children (WIC)?

____

…struggle with hunger?

____

…lack access to healthy foods at home?

____

20. Are the food and beverages for your center's meal service prepared on site?

- ◯ Yes
- ◯ No
- ◯ Both yes and no (*please explain*):

____

21. How are the food and beverages for your center's meal service primarily obtained?

- ◯ In-person shopping at a store
- ◯ Online ordered then picked up in-person
- ◯ Online and delivered
- ◯ Over the phone with a vendor

If you answered "In person shopping at store", or "Online ordered then picked up in-person", approximately how many miles to and from (roundtrip) does the center's food purchaser travel to get to the location where the center's foods and beverages are primarily obtained?

____

22. Who is responsible for planning meals for 3-5 year old children (*select all that apply*)?

- ☐ Owner of ECE
- ☐ Director or site supervisor/manager
- ☐ Family childcare provider
- ☐ Cook or chef
- ☐ Catering company
- ☐ Dietician
- ☐ Parents/guardians provide food for their children
- ☐ Other (*please specify*):

____

23. In which of the following does your center participate (*select all that apply*)?

- ☐ Child and Adult Care Food Program (CACFP) by USDA, which provides reimbursement for foods served
- ☐ Nutrition and Physical Activity Self-Assessment for Child Care (Go NAP SACC)
- ☐ Healthy Body, Healthy Minds
- ☐ Happy Healthy Homes
- ☐ Certified Early Childhood

24. How often does your center participate in out-of-center activities within the community (*for example, visiting public parks, eating at restaurants, nearby field trips, etc.*)?

- ◯ Very often
- ◯ Somewhat often
- ◯ Not very often
- ◯ Never

25. Please indicate which of the following are provided in your center for 3-5 year old children (*select all that apply*)?

- ☐ Breakfast
- ☐ Lunch
- ☐ Dinner
- ☐ Mid-morning snack
- ☐ Mid-afternoon snack
- ☐ Evening snack

26. Please indicate which of the following meals are provided by parents, and not by the center, for 3-5 year old children (*select all that apply*)?

- ☐ Breakfast
- ☐ Lunch
- ☐ Dinner
- ☐ Mid-morning snack
- ☐ Mid-afternoon snack
- ☐ Evening snack

27. Does your center currently have a health advisory committee? *(Note: a health advisory committee is a group of parents and/or community partners that meet to discuss ideas and programs to create healthier spaces for children at your center.)*

- ○ Yes
- ○ No
- ○ Not sure

28. Does your center have a policy restricting outdoor play during certain temperatures (*for example, if it is less than 50 degrees outside*)?

- ○ Yes: Oklahoma Child Care Licensing policy
- ○ Yes: Policy in addition to Oklahoma Child Care Licensing
- ○ No

If you answered "Yes: Policy in addition to OK CC Licensing", please specify:

____

CENTER ALLERGIES & ASTHMA

1. To your knowledge, what **estimated percentage** of 3-5 year old children in your center have asthma?

____

2. To your knowledge, what **estimated percentage** of 3-5 year old children in your center have wheezing or whistling in their chest?

____

3. To your knowledge, what **estimated percentage** of staff in your center have asthma?

____

4. Does your center have policies and guidelines for managing asthma medications?

- ○ Yes
- ○ No
- ○ Not sure

5. Does your center have policies and guidelines for reducing asthma allergens and irritants?

- ○ Yes
- ○ No
- ○ Not sure

6. Have you ever received any training on any of the following aspects of asthma (*select all that apply*)?

- ☐ No, I've never received any type of asthma training
- ☐ Asthma basics (causes of asthma, signs of asthma flare-ups)
- ☐ Reducing asthma allergens and irritants
- ☐ Asthma medication use and types
- ☐ Asthma management plans
- ☐ Proper administration of asthma medications

7. Does anyone who works at your center smoke or vape on your center's property?

- ○ Yes, but OUTSIDE only
- ○ Yes, both INSIDE and OUTSIDE
- ○ No

8. How confident are you in your center's ability to care for children with asthma?

- ◯ Not at all confident
- ◯ Slightly confident
- ◯ Somewhat confident
- ◯ Very confident
- ◯ Extremely confident

9. In the past 12 months, did you keep any pets such as cats, dogs, gerbils, or birds at your center?

- ◯ Yes
- ◯ No

10. Does your center have wall-to-wall carpet?

- ◯ Yes
- ◯ No

11. Which of the following, if any, are a concern to you at or around your center (*select all that apply*)?

- ☐ None – I do not have any concerns
- ☐ Indoor or outdoor air quality
- ☐ Lead
- ☐ Water quality
- ☐ Radon
- ☐ Crime
- ☐ Asthma and/or allergies
- ☐ Noise/noise pollution
- ☐ Housing quality
- ☐ Natural disasters
- ☐ Toxins in pesticides and/or cleaners
- ☐ Other issues (*please specify*):

____

CLASSROOM HEALTH PRACTICES: NUTRITION

Please read each statement or question carefully and check the response that best fits your child care facility. Refer to the instructions at the beginning of this survey for clarification on how to complete survey items.

*As a reminder, these questions ask about health practices in your preschool-age classroom(s) (**3-5 year old**) or Head Start program, NOT infant classroom or your Early Head Start program (if you have one). Please answer questions about your preschool classroom **with children ages 3-5 years**.*

Fruits and Vegetables Served				
1. Fruit (not juice) is offered:	◯ 3 times per week or less	◯ 4 times per week	◯ 1 time per day	◯ 2 or more times per day
2. Fruit is offered canned **in own juice** (no syrups), fresh, or frozen:	◯ Rarely or never	◯ Some of the time	◯ Most of the time	◯ All of the time
3. Vegetables (not including French fries, tater tots, hash browns or dried beans) are offered:	◯ 2 times per week or less	◯ 3-4 times per week	◯ 1 time per day	◯ 2 or more times per day
4. Vegetables, other than potatoes, corn, and green beans are offered:	◯ Less than 1 time per week	◯ 1-2 times per week	◯ 3-4 times per week	◯ 1 or more times per day
5. Cooked vegetables are prepared with added meat fat, margarine or butter:	◯ All of the time	◯ Most of the time	◯ Some of the time	◯ Rarely or never

Meats, Fats, and Grains				
6. Fried or pre-fried potatoes (French fries, tater tots, hash browns) are offered:	◯ 3 or more times per week	◯ 2 times per week	◯ 1 time per week	◯ Less than once a week or never
7. Fried or pre-fried (frozen and breaded) meats or fish (chicken nuggets, fish sticks) are offered:	◯ 3 or more times per week	◯ 2 times per week	◯ 1 time per week	◯ Less than once a week or never
8. High fat meats (sausage, bacon, hot dogs, bologna, ground beef) are offered:	◯ 3 or more times per week	◯ 2 times per week	◯ 1 time per week	◯ Less than once a week or never
9. Beans or lean meats (baked or broiled chicken, turkey, or fish) are offered:	◯ Less than 1 time per week	◯ 1-2 times per week	◯ 3-4 times per week	◯ 1 or more times per day
10. High fiber, whole grain foods (whole wheat bread, oatmeal, brown rice, Cheerios, etc.) are offered:	◯ 1 time per week or less	◯ 2-4 times per week	◯ 1 time per day	◯ 2 or more times per day
11. Sweets or salty foods (cookies, cakes, muffins, chips, etc.) are offered:	◯ 1 or more times per day	◯ 3-4 times per week	◯ 1-2 times per week	◯ Less than once a week or never

Beverages				
12. Drinking water outside is:	◯ Not visible	◯ Visible and available during designated water breaks	◯ Easily visible and available on request	◯ Easily visible and available for self-serve
13. Drinking water inside is:	◯ Not visible	◯ Visible and available during designated water breaks	◯ Easily visible and available on request	◯ Easily visible and available for self-serve
14. <u>100% </u>fruit juice is offered:	◯ 2 or more times per day	◯ 1 time per day	◯ 3-4 times per week	◯ 2 times per week or less
15. Sugary drinks (Kool-Aid, sports drinks, sweet tea, punches, soda) other than 100% juice are offered:	◯ 1 or more times per week	◯ Less than 1 time per week	◯ Less than 1 time per month	◯ Rarely or never
16. Milk served to children ages 3 years and older is usually:	◯ Whole or regular	◯ 2% reduced fat	◯ 1% low fat	◯ Skim or non-fat
17. Soda and other vending machines are located:	◯ In entrance or front of building	◯ In public areas, but not entrance	◯ Out of sight of parents and children	◯ No vending machines on site

Menus and Variety				
18. Menus used are:	◯ 1-week cycle	◯ 2-week cycle	◯ 3-week cycle or more without seasonal change	◯ 3-week cycle or more with seasonal change

19. Weekly menus include a combination of both new and familiar foods:	◯ Rarely or never	◯ Some of the time	◯ Most of the time	◯ All of the time
20. Weekly menus include foods from a variety of cultures:	◯ Rarely or never	◯ Some of the time	◯ Most of the time	◯ All of the time

Feeding Practices				
21. When children eat less than half of a meal or snack, the staff help determine if they are full before removing the plate:	◯ Rarely or never	◯ Some of the time	◯ Most of the time	◯ All of the time
22. When children request seconds, staff help determine if they are still hungry before serving additional food:	◯ Rarely or never	◯ Some of the time	◯ Most of the time	◯ All of the time
23. Children are encouraged by staff to try a new or less favorite food:	◯ Rarely or never	◯ Some of the time	◯ Most of the time	◯ All of the time
24. Food is used to encourage positive behavior:	◯ All of the time	◯ Most of the time	◯ Some of the time	◯ Rarely or never

Foods Offered Outside of Regular Meals and Snacks				
25. Guidelines provided to parents for food brought in for holidays or celebrations are:	◯ Not available	◯ Loose guidelines with healthier options encouraged	◯ Written guidelines for healthier options that are not always enforced	◯ Written guidelines for healthier options that are usually enforced
26. Holidays are celebrated with mostly healthy foods or with non-food treats like stickers:	◯ Rarely or never	◯ Some of the time	◯ Most of the time	◯ All of the time
27. Fundraising consists of selling only non-food items (like wrapping paper, coupon books or magazines):	◯ Rarely or never	◯ Some of the time	◯ Most of the time	◯ All of the time

Supporting Healthy Eating				
28. Staff join children at the table for meals:	◯ Rarely or never	◯ Some of the time	◯ Most of the time	◯ All of the time
29. Meals are served family style (children serve themselves with limited help):	◯ Rarely or never	◯ Some of the time	◯ Most of the time	◯ All of the time
30. Staff consume the same food and drinks as the children:	◯ Rarely or never	◯ Some of the time	◯ Most of the time	◯ All of the time
31. Staff eat or drink less healthy foods (especially sweets, soda and fast food) in front of the children:	◯ All of the time	◯ Most of the time	◯ Some of the time	◯ Rarely or never

32. Staff talk informally with children about trying and enjoying healthy foods:	◯	Rarely or never	◯	Some of the time	◯	Most of the time	◯	All of the time
33. Support for good nutrition is visibly displayed in 3 to 5 year old classrooms and common areas by:	◯	No posters, pictures, or books about healthy food displayed	◯	A few posters, pictures, or books about healthy food displayed in a few rooms	◯	Posters, pictures, or books about healthy food displayed in most rooms	◯	Posters, pictures, or books about healthy food displayed in every room

Nutrition Education for Staff, Children, and Parents								
34. Training opportunities on nutrition (other than food safety and food program guidelines) are provided for staff:	◯	Rarely or never	◯	Less than 1 time per year	◯	1 time per year	◯	2 times per year or more
35. Nutrition education is provided for children through a standardized curriculum:	◯	Rarely or never	◯	1 time per month	◯	2-3 times per month	◯	1 time per week or more
36. Nutrition education opportunities are offered to parents (workshops, activities and take-home materials):	◯	Rarely or never	◯	Less than 1 time per year	◯	1 time per year	◯	2 times per year or more

Nutrition Policy								
37. A written policy on nutrition and food service that covers most of the above topics:	◯	Does not exist	◯	Exists informally, but is not written or followed	◯	Is written, but not always followed	◯	Is written, available and followed

<u>BARRIERS TO CLASSROOM HEALTH PRACTICES: NUTRITION</u>

The list below includes possible barriers which some ECE centers and teachers feel makes it harder to ***serve healthier meals and snacks****, such as those suggested by the Child and Adult Care Food Program (CACFP) best practices. Examples of those best practices include the following:*

- *Include fruits and vegetables as snacks*
- *Serve no juice*
- *Include vegetable subgroups throughout the week*
- *Serve meals family style*
- *Serve 2 servings of whole grain food per day*

There are no right or wrong answers. For each statement, please respond "YES" if you feel this is a barrier which your center faces or not, specific to ***serving healthier meals and snacks for 3-5 year old children****.*

	YES	NO
1. Not enough money to cover the cost of serving healthier meals and snacks	◯	◯
2. Lack of control over the types of meals and snacks that are delivered to us	◯	◯
3. Those preparing meals and snacks lack the knowledge to prepare healthier foods and beverages	◯	◯
4. Those preparing meals and snacks lack the time to prepare healthier foods and beverages	◯	◯

	YES	NO
5. Children would not like the taste of healthier meals and snacks	◯	◯
6. Directors/providers are concerned about wasting food because children won't eat healthier meals and snacks	◯	◯
7. Parents/guardians do not want children to be served healthier foods	◯	◯
8. Parents/guardians provide unhealthy snacks and meals	◯	◯
9. Limited space for food storage, such as refrigerator and cabinet space	◯	◯
10. Lack of availability of healthy foods in my area	◯	◯
11. Lack of support from other providers	◯	◯
12. Other areas in our program have higher priority than nutrition at this time	◯	◯
13. So many different recommendations that providers do not know which to follow	◯	◯
14. Unsure which foods can be reimbursed through CACFP	◯	◯
15. Weekly schedule limits time to shop more than once per week	◯	◯

16. Please describe any other barriers not listed above:

____

The list below includes possible barriers which some ECE centers and teachers feel makes it harder to ***use healthful mealtime practices****, such as those suggested by the Child and Adult Care Food Program (CACFP) best practices. Examples of those best practices include the following:*

- *Praising children for trying new foods*
- *Talking with children about healthy foods*
- *Allowing children to decide when they are full*
- *Sitting with children during mealtime and eating the same foods*
- *Serving meals family style*

There are no right or wrong answers. For each statement, please respond "YES" if you feel this is a barrier which your center faces or not, specific to ***using healthful mealtime practices for 3-5 year old children****.*

	YES	NO
1. Providers do not have time to sit with children during meals	◯	◯
2. There are not enough providers in the program to sit with children during meals	◯	◯
3. There is not enough money to cover the cost of serving meals and snacks to providers	◯	◯
4. Providers are unsure how to encourage children's healthy eating	◯	◯
5. Providers do not like the taste of the healthy foods that are served at the childcare program, so they have trouble encouraging children's healthy eating at mealtime	◯	◯
6. Providers have dietary restrictions, so they find It difficult to eat the same foods that are served to children	◯	◯
7. Providers are uncertain how to handle children who are hesitant to try new foods	◯	◯
8. Providers feel mealtimes with children are stressful/chaotic	◯	◯
9. If you let children serve themselves, they would **not eat/drink enough**	◯	◯
10. If you let children serve themselves, they would **eat/drink too much**	◯	◯
11. If you let children serve themselves, they will make too much of a mess	◯	◯

12. Please describe any other barriers not listed above:

____

CLASSROOM HEALTH PRACTICES: PHYSICAL ACTIVITY

Please read each statement or question carefully and check the response that best fits your child care facility. Refer to the instructions at the beginning of this survey for clarification on how to complete survey items.

*As a reminder, these questions ask about health practices in your preschool-age classroom(s) (**3-5 year old**) or Head Start program, NOT infant classroom or your Early Head Start program (if you have one). Please answer questions about your preschool classroom **with children ages 3-5 years**.*

Active Play and Inactive Time				
1. Active play time is provided to all children:	◯ 45 minutes or less each day	◯ 46-90 minutes each day	◯ 91-120 minutes each day	◯ More than 120 minutes each day
2. Teacher-led physical activity is provided to all children:	◯ 1 time per week or less	◯ 2-4 times per week	◯ 1 time per day	◯ 2 or more times per day
3. Outdoor active play is provided for all children:	◯ 1 time per week or less	◯ 2-4 times per week	◯ 1 time per day	◯ 2 or more times per day
4. Active play time is withheld for children who misbehave:	◯ Often	◯ Sometimes	◯ Never	◯ Never and we provide more active play time for good behavior
5. Children are seated (excluding naps and meals) more than 30 minutes at a time:	◯ 1 or more times per day	◯ 3-4 times per week	◯ 1-2 times per week	◯ Less than once a week or never
6. Television and video use consists of the:	◯ TV turned on for 5 or more hours per week	◯ TV turned on for 3-4 hours per week	◯ TV turned on for 2 hours per week or less	◯ TV used rarely or never

Play Environment				
7. Fixed play equipment (tunnels) balancing equipment, climbing equipment, overhead ladders) is:	◯ Unavailable at our site	◯ Only one type of equipment is available	◯ Different equipment available that suits most children	◯ Wide variety of equipment available and accommodates needs of all children
8. Portable play equipment (wheel toys, balls, hoops, ribbons) consists of:	◯ Little variety and children must take turns	◯ Some variety but children must take turns	◯ Good variety but children must take turns	◯ Lots of variety for children to use at same time
9. Outdoor portable play equipment is:	◯ Available during special times only	◯ Located out of child sight and reach, staff must access	◯ Available on request	◯ Freely available by children at all times
10. Outdoor space includes:	◯ No open running spaces, no track/path for wheeled toys	◯ Very limited open running space, no track/path for wheeled toys	◯ Plenty of open running space, no track/path for wheeled toys	◯ Plenty of open running spaces and a track/path for wheeled toys

11. Indoor play space is available:	◯ For quiet play only	◯ For very limited movement (jumping and rolling)	◯ For some active play (jumping, rolling and skipping)	◯ For all activities, including running

Supporting Physical Activity				
12. During active play time staff:	◯ Supervise play only (mostly sit or stand)	◯ Sometimes encourage children to be active	◯ Sometimes encourage children to be active and join children in active play	◯ Often encourage children to be active and join children in active play
13. Support for physical activity is visibly displayed in 3 to 5 year old classrooms and common areas by:	◯ No posters, pictures, or books about physical activity displayed	◯ A few posters, pictures, or books about physical activity displayed in a few rooms	◯ Posters, pictures, or books about physical activity are displayed in most rooms	◯ Posters, pictures, or books about physical activity are displayed in every room

Physical Activity Education for Staff, Children, and Parents				
14. Training opportunities are provided for staff in physical activity (not including playground safety):	◯ Rarely or never	◯ Less than 1 time per year	◯ 1 time per year	◯ 2 times per year or more
15. Physical activity education (motor-skill development) is provided for children through a standardized curriculum:	◯ Rarely or never	◯ 1 time per month	◯ 2-3 times per month	◯ 1 time per week or more
16. Physical activity education is offered to parents (workshops, activities and take-home materials):	◯ Rarely or never	◯ Less than 1 time per year	◯ 1 time per year	◯ 2 times per year or more

Physical Activity Policy				
17. A written policy on physical activity that covers most of the above topics:	◯ Does not exist	◯ Exists informally, but is not written or followed	◯ Is written, but not always followed	◯ Is written, available and followed

BARRIERS TO CLASSROOM HEALTH PRACTICES: PHYSICAL ACTIVITY

The list below includes possible barriers which some ECE centers and teachers feel makes it harder to ***promote physical activity****. Examples of those practices include the following:*

- *Providing indoor and outdoor active playtime*
- *Talking with children about physical activity*
- *Providing teacher-led physical activity*
- *Providing indoor and outdoor play space and equipment*
- *Verbally and physically encouraging children to be physically active*

There are no right or wrong answers. For each statement, please respond "YES" if you feel this is a barrier which your center faces or not, specific to ***promoting physical activity for 3-5 year old children****.*

	**YES**	**NO**
1. There are competing curriculum priorities that take precedent over promoting physical activity	◯	◯
2. Providers are unsure how to encourage children's participation in physical activity	◯	◯
3. Limited space for storing activity-promoting toys and equipment	◯	◯
4. Lack of resources to purchase activity-promoting toys and equipment	◯	◯
5. Limited room for indoor active playtime	◯	◯
6. Limited room for outdoor active playtime	◯	◯
7. Undesirable weather conditions that do not permit outdoor playtime	◯	◯
8. Students are reluctant to participate in physical activity	◯	◯
9. The school board does not support the idea of promoting physical activity for children	◯	◯
10. Parents/guardians do not support the idea of promoting physical activity for children	◯	◯
11. Children often arrive to school wearing clothing that is inappropriate for outdoor play or improperly fitting	◯	◯
12. Providers are concerned children will injure themselves during active playtime	◯	◯
13. Providers are concerned about neighborhood safety during active playtime	◯	◯
14. Playground licensing requirements limit ability to provide equipment that allows for vigorous physical activity	◯	◯
15. Providers prefer to remain indoors and/or partake in sedentary classroom activities over physical activities	◯	◯
16. Providers feel playtime with children is stressful/chaotic	◯	◯

17. Please describe any other barriers not listed above:

____

CENTER CLEANERS AND AIR FRESHENERS

1. How often do you use bleach at your center?
- ◯ Never, we do not use bleach
- ◯ Daily or a few times a day
- ◯ Weekly or a few times a week
- ◯ Monthly or a few times a month
- ◯ Every few months or less often

2. Does your center use low toxicity or less toxic cleaners?
- ◯ Yes
- ◯ No
- ◯ Not sure

3. What kind of air fresheners are used at your center (*select all that apply*)?
- ☐ None – do not use any type of air freshener or candle
- ☐ Scented candles
- ☐ Spray air fresheners
- ☐ Continuous release (like a plug-in)
- ☐ Incense
- ☐ Essential oils (reed diffuser or other type of diffuser)
- ☐ Essential oil electric or battery diffuser
- ☐ Potpourri
- ☐ Gel canister
- ☐ Other types of air freshener(s) (*please specify*):

  ____

CENTER PESTS AND PEST CONTROL

1. To your knowledge, what year was your center building originally built?

____

2. Integrated Pest Management, often called "IPM" for short, is an approach to keeping pests (bugs, weeds, rats, etc.) below harmful levels and reducing or eliminating pesticide use. Have you heard of IPM?
- ◯ Yes
- ◯ No

3. Does your center use Integrated Pest Management ("IPM") strategies for pest control?
- ◯ Yes
- ◯ No
- ◯ Not sure

4. Does your center have a written policy for use of pesticides (bug killers, weed killers, rat killers, etc.), stating when and how to apply them?
- ◯ Yes
- ◯ No
- ◯ Not sure
- ◯ Not applicable, **no** pesticides are used

5. Are staff notified before pesticides (including weed killers) are going to be applied **INSIDE or OUTSIDE** of your center?
- ◯ Yes
- ◯ No
- ◯ Not applicable, **no** pesticides are used

6. Are parents notified before pesticides (including weed killers) are going to be applied **INSIDE or OUTSIDE** of your center?
- ◯ Yes
- ◯ No
- ◯ Not applicable, **no** pesticides are used

THE FOLLOWING QUESTIONS ASK ABOUT PESTS AND PEST CONTROL METHODS **INSIDE** YOUR CENTER.

7. In the past 12 months, which of the following pests were a problem **INSIDE** your center (i.e., INDOORS) *(select all that apply)*?

- ☐ None- **did not** have any **INDOOR** pest problems.
- ☐ Ants ☐ Fleas ☐ Termites ☐ Snails/slugs ☐ Mold
- ☐ Cockroaches ☐ Rodents ☐ Aphids ☐ Spiders ☐ Head lice
- ☐ Bed bugs ☐ Other pest(s) *(please specify):* ______________________

8. In the past 12 months, which of the following pest control methods did your center use for controlling pests **INSIDE** your center (i.e., INDOORS) *(select all that apply)*?

- ☐ Nothing used ☐ Sprayed Pesticides ☐ Bait stations or poison traps
- ☐ Sticky fly strips ☐ Mouse or rat traps ☐ Moth balls
- ☐ Poison pellets or powders ☐ Removed food sources ☐ Fixed leaks
- ☐ Cleaned the area ☐ Sealed cracks/openings ☐ Installed screens or other barriers
- ☐ Other *(please specify):* ______________________

9. In the past 12 months, who applied the **INDOOR** pesticides used at your center *(select all that apply)*?

- ☐ Nothing used
- ☐ Myself ☐ Director ☐ Another staff member
- ☐ Property owner ☐ Custodial/janitorial staff ☐ Pest control company
- ☐ Not sure ☐ Other *(please specify):* ______________________

10. In the past 12 months, how frequently were pesticides sprayed, scattered, or "bombed" **INSIDE** your center (i.e., INDOORS) *(select all that apply)*?

- ☐ Nothing used ☐ Once a week ☐ Once a month
- ☐ Once a year ☐ A few times a year ☐ Whenever pests become a problem
- ☐ Not applicable- pesticides were used, **but not** sprayed, scattered, or "bombed"

THE FOLLOWING QUESTIONS ASK ABOUT PESTS AND PEST CONTROL METHODS **OUTSIDE** YOUR CENTER.

11. In the past 12 months, which of the following pests were a problem **OUTSIDE** your center (i.e., OUTDOORS) *(select all that apply)*?

- ☐ None- **did not** have any **OUTDOOR** pest problems.
- ☐ Ants ☐ Fleas ☐ Termites ☐ Snails/slugs ☐ Mold
- ☐ Cockroaches ☐ Rodents ☐ Aphids ☐ Spiders ☐ Scorpions
- ☐ Wasps/yellow jackets ☐ Other pest(s) *(please specify):* ______________________

12. In the past 12 months, which of the following pest control methods did your center use for controlling pests **OUTSIDE** your center (i.e., OUTDOORS) *(select all that apply)*?

- ☐ Nothing used
- ☐ Sprayed Pesticides
- ☐ Bait stations or poison traps
- ☐ Sticky fly strips
- ☐ Mouse or rat traps
- ☐ Applied weed killer
- ☐ Poison pellets or powders
- ☐ Removed food sources
- ☐ Fixed leaks
- ☐ Cleaned the area
- ☐ Sealed cracks/openings
- ☐ Installed screens or other barriers
- ☐ Cut grass or weeds
- ☐ Other pest(s) *(please specify):* ______________________

13. In the past 12 months, who applied the **OUTDOOR** pesticides used at your center *(select all that apply)*?

- ☐ Nothing used
- ☐ Myself
- ☐ Director
- ☐ Another staff member
- ☐ Property owner
- ☐ Custodial/janitorial staff
- ☐ Pest control company
- ☐ Not sure
- ☐ Other *(please specify):* ______________________

14. In the past 12 months, how frequently were pesticides sprayed, scattered, or "bombed" **OUTSIDE** your center (i.e., OUTDOORS) *(select all that apply)*?

- ☐ Nothing used
- ☐ Once a week
- ☐ Once a month
- ☐ Once a year
- ☐ A few times a year
- ☐ Whenever pests become a problem
- ☐ Not applicable- pesticides were used, **but not** sprayed, scattered, or "bombed"

Thank you! We greatly appreciate the time you have taken to complete this survey. For your convenience, **please use the postage-paid return envelope included in your survey packet to return your questionnaire**.

Finally, if you would like to be entered into a drawing for one of forty-five $25 Amazon gift cards, **please include your name and email address below**; this information will **not** be connected with your survey responses, and will **only** be used to contact those who have been randomly selected to receive Amazon gift cards.

Name: ____

Email Address: ____

If you have any questions concerning the research study, please contact the Principal Investigator, Dr. Susan Sisson at susan-sisson@ouhsc.edu (405-271-2113 x 41176) or Study Coordinator, Bethany Williams at Bethany-Williams@ouhsc.edu (405-271-2113 x 41173).
